# Investigating Alcohol Sweetspot Phenomena in Reduced Alcohol Red Wines

**DOI:** 10.3390/foods8100491

**Published:** 2019-10-14

**Authors:** Duc-Truc Pham, Vanessa J. Stockdale, David W. Jeffery, Jonathan Tuke, Kerry L. Wilkinson

**Affiliations:** 1 School of Agriculture, Food and Wine, The University of Adelaide, Waite Campus, PMB 1, Glen Osmond 5064, Australia; duc-truc.pham@adelaide.edu.au (D.-T.P.); david.jeffery@adelaide.edu.au (D.W.J.); 2 The Australian Research Council Training Centre for Innovative Wine Production, PMB 1, Glen Osmond 5064, Australia; Vanessa.Stockdale@tweglobal.com; 3 Treasury Wine Estates, 97 Sturt Highway, Nuriootpa 5352, Australia; 4 School of Mathematical Sciences, The University of Adelaide, Adelaide 5000, Australia; simon.tuke@adelaide.edu.au

**Keywords:** alcohol adjustment, evaporative perstraction, one proportion test, partial dealcoholization, reverse osmosis, sensory analysis

## Abstract

Warmer growing seasons, variations to grape ripening dynamics, and stylistic changes have contributed to increased wine alcohol levels, which can negatively impact sensory properties. As a consequence, winemakers have sought technological innovations to produce reduced alcohol wine (RAW). The sensory methodology used by industry to optimize the ethanol content of RAW is known as ‘alcohol sweetspotting’. However, to date, there is no scientific evidence to support the alcohol sweetspot phenomenon, and the sensory methodology used for alcohol sweetspotting has not been validated. In this study, different methods of presenting wine samples (i.e., ordered vs. randomized, and linear vs. circular) were employed to determine to what extent presentation order influences the outcome of alcohol sweetspotting trials. Two different approaches to statistical analysis of sensory data, i.e., chi-square goodness of fit vs. one proportion tests, were also evaluated. Statistical analyses confirmed alcohol sweetspots were apparent in some sweetspot determination trials, but outcomes were not reproducible in replicate determinations (either by panel or by individual panelists). Analysis of data using the one proportion test improved the likelihood of identifying statistically significant differences between RAWs, but variation in individuals’ sensitivity to differences in sensory properties following ethanol removal prevented validation of the alcohol sweetspot phenomenon based on the wines studied.

## 1. Introduction

The alcohol content of wine has steadily increased in recent years [1], in part due to the warmer temperatures experienced during the growing season, as a consequence of climate change. This has financial implications for winemakers, not only because wines with higher alcohol levels attract higher import duties and taxes, but because wine producers and consumers alike, are increasingly mindful of the health and social issues associated with excessive alcohol consumption [2]. The wine industry is therefore adopting various strategies for correcting the alcohol content of wine, including the partial dealcoholization of wine using reverse osmosis-evaporative perstraction (RO-EP) [3]. This process employs membrane technologies to fractionate wine and remove ethanol from the resulting permeate, before the (partially) dealcoholized permeate is recombined with the retentate, to give reduced alcohol wine (RAW). Partial dealcoholization of wine has also be achieved using pervaporation and spinning cone column distillation technologies [4,5]. It is still unclear, however, how to determine optimal ethanol levels for RAW from a sensory perspective.

Some wine producers employ a process referred to as ‘alcohol sweetspotting’ to optimize wine alcohol levels [6]. This involves partial dealcoholization of wine (by RO-EP) and subsequent blending of the base wine and RAW to generate a series of wines (typically 8–10) comprising alcohol levels that differ by small, incremental amounts (e.g., 0.2% alcohol by volume (abv)). The blends are then evaluated (typically in ascending or descending order of ethanol concentration) by three or four winemakers, who identify the wine (or wines) they considered to exhibit superior organoleptic properties (i.e., optimal aroma, flavor, taste, mouthfeel and balance). To date, however, the existence of an alcohol sweetspot phenomenon has not been scientifically validated.

The impacts of ethanol on wine sensory properties tend to support the concept of an alcohol sweetspot. Depending on its concentration, ethanol can affect the perception of taste and mouthfeel properties by enhancing or suppressing sweetness, sourness, bitterness, saltiness, astringency and hotness [7,8,9,10]. Moreover, ethanol can enhance wine aroma by masking undesirable attributes and/or harmonising imbalances, but the perception of desirable aromas and flavors can also be diminished by ethanol [11,12]. At lower levels. i.e., 5–12% abv, ethanol facilitates the release of volatile compounds under dynamic conditions and maintains their headspace concentrations to enhance wine aroma [13]. Conversely, higher ethanol levels, i.e., 10–18% abv, are negatively correlated with volatile headspace concentrations, reflecting the increased solubility of volatiles in ethanol, relative to water [14].

Several previous studies have attempted to validate the alcohol sweetspot phenomenon. A 2013 study investigated alcohol sweetspots in three white wines and a red wine, using an expert panel [15]. The ethanol content of the wines evaluated via ranking and triangle tests ranged by 3% abv, spaced at 0.5% intervals, e.g., 12.1, 12.6, 13.1, 13.6, 14.1, 14.6 and 15.1% abv for a bracket of Riesling wines. Panelists ranked wines according to their individual preferences, with the most preferred wine being ranked 1 and the least preferred wine ranked 7. Each bracket was evaluated in triplicate, using three different orders of presentation: increasing ethanol content; decreasing ethanol content; and a randomized presentation order. Although there were no clear preferences for wines based on alcohol content, wines with ethanol levels above 15% abv were consistently ranked lower than other samples. It is not clear to what extent the spacing between wine ethanol levels (i.e., 0.5%) may have influenced the identification of any alcohol sweetspots.

In a subsequent study, consumers evaluated subsets of Chardonnay wine comprising samples for which the ethanol content had been adjusted to span a concentration range of approximately 1% abv (at 0.2% increments), using a wine that was partially dealcoholized by spinning cone column distillation [16]. Consumers were randomly allocated to wine subsets and asked to rate their liking of samples using a 9-point hedonic category scale, but only one consumer group yielded results (i.e., a positive quadratic curve) that suggested the existence of an alcohol sweetspot (being 13.8 to 14.0% abv) for their subset of wines. Consistent liking scores were not obtained for other wine subsets and therefore did not enable identification of a sweetspot for that wine.

The alcohol sweetspotting process is likely affected by a combination of factors, including the order in which samples are presented, the incremental difference in alcohol levels between samples, and the composition of the sensory panel (i.e., consumers vs. winemakers/experts). Standardized sensory methodology typically requires randomized presentation of samples, whereas industry based sweetspotting trials usually present samples in sequential order (i.e., increasing or decreasing ethanol concentration [5]). Randomization of samples may confound the perception of subtle differences between samples of similar alcohol content, whereas sequential presentation of samples may introduce bias (i.e., away from the samples of highest and/or lowest alcohol content).

This study aimed to investigate to what extent different methods of presenting samples (i.e., ordered vs. randomized, and linear vs. circular) might influence the outcome of alcohol sweetspotting trials. These approaches were intended to evaluate the ability of panelists to choose the same sample in replicate brackets, as well as to investigate whether the order in which samples were assessed influenced the sensory perceptions of wines. Different approaches to statistical analysis of data from sweetspotting trials were also evaluated, i.e., chi-square tests for ‘goodness of fit’ vs. ‘one proportion’, in an attempt to further provide scientific evidence of an alcohol sweetspot.

## 2. Materials and Methods

### 2.1. Wine Samples

Three 2015 Barossa Valley red wines, a Shiraz Cabernet Sauvignon blend (60:40) and two Shiraz wines, hereafter wines A, B and C, respectively, were sourced from a commercial winery who had deemed the wines to be in need of alcohol adjustment. The wines were partially dealcoholized using an industrial scale RO-EP unit (VA Filtration, Nuriootpa, Australia), in accordance with manufacturer operating instructions [3]; their alcohol concentrations before and after RO-EP treatment were: 16.0 and 14.4% abv for wine and RAW A; 16.0 and 14.2% abv for wine and RAW B; and 16.3 and 14.0% abv for wine and RAW C.

Wines A and B were subsequently blended with different proportions of their corresponding RAW to generate a series of samples with ethanol concentrations that differed by 0.2% abv, for use in alcohol sweetspotting trials, according to the practices typically employed by the winery. Trial 1 comprised nine blends of wine A and RAW A, with alcohol percentage levels: 16.0 (wine A), 15.8, 15.6, 15.4, 15.2, 15.0, 14.8, 14.6, and 14.4 (RAW A); while Trial 2 comprised nine blends of wine B and RAW B, with alcohol percentage levels: 16.0 (wine B), 15.8, 15.6, 15.4, 15.2, 15.0, 14.8, 14.6, and 14.2 (RAW B). Wine C was blended with its corresponding RAW to achieve samples which differed in ethanol concentration by 0.2, 0.5 and 1.0% abv from both wine C (i.e., 16.1, 15.8 and 15.3% abv) and RAW C (i.e., 14.2, 14.5 and 15.0% abv), for use in difference tests.

Wines, RAWs and blends thereof were subsequently bottled (under screw cap) in 750 mL glass bottles and cellared (in darkness at 15 °C) prior to chemical and sensory analyses (performed approximately 1 month after RO-EP treatment, blending and bottling).

### 2.2. Sensory Analysis of Wines

For each of the sensory analyses performed, i.e., alcohol sweetspotting trials and difference tests: samples (30 mL) were served at ambient temperature (i.e., 22–24 °C), in covered, three-digit coded XL5 wine glasses (International Organization for Standardization, ISO 3591:1977), under LED white lighting. Alcohol sweetspotting trials were held in an open-plan sensory laboratory (with each panelist on a separate bench) at a commercial winery in the Barossa Valley wine region. Difference tests were held in a sensory laboratory at the University of Adelaide’s Waite Campus. Sensory trials were approved by the Human Research Ethics Committee of the University of Adelaide (H-2015-094).

#### 2.2.1. Alcohol Sweetspotting Trials

Expert panels, each comprising 14 winemakers, were assembled for alcohol sweetspotting trials. Prior to evaluation, each panelist completed a short survey comprising demographic questions. For each trial, a higher proportion of panelists were male (64–79%) and aged between 31 and 50 years (72–85%), but the majority had more than 10 years industry experience, all had wine judging experience, and 79% had previously undertaken alcohol sweetspotting trials (Table S1).

**Figure 1:**
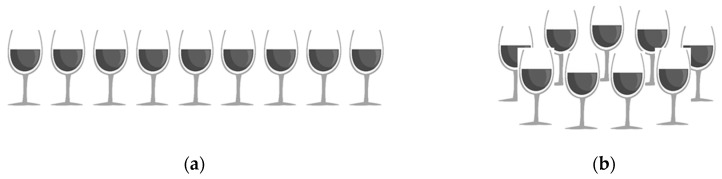
(**a**) Linear and (**b**) circular presentation formats used in alcohol sweetspotting trials.

Panelists were presented with brackets of nine samples at a time (comprising wine, RAW and blends thereof), using four different orders of presentation: (i) samples presented in a linear format, in random order, hereafter ‘row, randomized’; (ii) samples presented in a linear format, with alcohol content decreasing from left to right, hereafter referred to as ‘row, ordered’; (iii) samples presented in a circular format (i.e., evenly spaced around a circular tray), in random order, hereafter ‘circular, randomized’; and (iv) samples presented in a circular format with alcohol content increasing in a clockwise direction, hereafter referred to as ‘circular, ordered’ (Figure 1).

Panelists were instructed to evaluate samples presented in rows from left to right, whereas for samples presented in a circle, panelists were instructed to evaluate samples in a clockwise direction, starting with any sample. For each trial, panelists evaluated eight brackets of samples in total, i.e., four presentation orders in duplicate, with brackets also being presented in a randomized order. Short breaks (2 min) were enforced between brackets, with longer breaks (30–60 min) enforced after the first four brackets. In Trial 1, panelists were asked to identify one or more samples from each bracket that they considered exhibited superior overall sensory properties; but in Trial 2, the panel was asked to identify only one sample. Trials 1 and 2 were held on different days.

#### 2.2.2. Difference Tests

A series of triangle tests [17] were conducted with a panel of 18 assessors comprising wine science staff and students from the University of Adelaide (nine males and nine females, aged between 18 and 55 years) to determine the change in ethanol concentration that resulted in perceptible differences in sensory properties for wine C and RAW C (using blends thereof, prepared as outlined above). Samples were presented in a balanced, randomized presentation order comprising all possible configurations (i.e., XXY, XYX, YXX, YYX, YXY and XYY, where X denotes wine C or RAW C, and Y denotes blends that differed in ethanol concentration by 0.2, 0.5 and 1.0% abv), an equal number of times. Panelists were asked to smell and taste the samples presented in each bracket, and to identify the sample that was different. Sensory data were collected and processed using RedJade online software (RedJade, Redwood Shores, CA, USA).

### 2.3. Chemical Analysis of Wines

The alcohol content, density, pH, titratable acidity (TA, as g/L of tartaric acid equivalents, to an endpoint of pH 8.2) and volatile acidity (VA, as g/L of acetic acid equivalents) were determined (in duplicate) by the Australian Wine Research Institute’s (AWRI) Commercial Services laboratory (Adelaide, Australia), using a Foss WineScan analyzer (Mulgrave, Australia). Glucose, fructose, glycerol and organic acids were determined (in duplicate) by high-performance liquid chromatography (HPLC), as described previously [18]. Briefly, an Agilent 1100 series HPLC (Agilent Technologies, Forest Hill, Australia), fitted with diode array and refractive index detectors was used, with separation achieved using an Aminex HPX-87H cation exchange column (Bio-Rad Laboratories, Gladesville, NSW, Australia) and 2.5 mM sulfuric acid as the mobile phase. Calibration curves relating concentrations to optical density or refractive index were fitted using ChemStation software (Agilent Technologies). Wine color measurements, including CIELab, were performed (in duplicate) using a Cintra 4040 spectrophotometer (GBC Scientific Equipment, Melbourne, Australia). Samples were filtered through 0.45 µm filters (Acrodisc, Sigma-Aldrich, Castle Hill, Australia) after which absorbance was recorded at 420, 520 and 800 nm. Wine color density and hue were calculated as: color density (au) = (A_520_ – A_800_) + (A_420_ – A_800_) and hue = (A_420_ – A_800_)/(A_520_ – A_800_) [19]. CIELab measurements determined L*, a* and b*, being coordinate values corresponding to the degree of lightness, and the intensity of red (when a* > 0), green (when a* < 0), yellow (when b* > 0) and blue (when b* < 0) hues [19].

The concentrations of several fermentation volatiles (acids, alcohols and esters) were determined (without replication, i.e., *n* = 1) by Metabolomics Australia (AWRI) using an Agilent 7890A gas chromatograph, equipped with a Gerstel MPS2 multipurpose autosampler and coupled to an Agilent 5875C mass selective detector, and previously reported stable isotope dilution analysis (SIDA) methods [20]. Headspace solid phase micro-extraction (HS-SPME) sampling of diluted wine (1 in 10 dilution in water) was performed in a 20 mL vial containing 2 g of sodium chloride, with the SPME fiber being exposed to the headspace for 10 min prior to desorption. Separation was achieved using a Phenomenex ZB-Wax column (60 m × 0.25 mm i.d. × 0.25 µm film thickness) and helium as the carrier gas (2.0 mL/min in constant flow mode). Preparation of isotopically labelled internal standards, method validation and instrument operating conditions are described extensively in the aforementioned publication [20].

### 2.4. Statistical Analysis

Basic compositional data were analyzed by one-way analysis of variance (ANOVA) using GenStat (15th Edition, VSN International Limited, Herts, England, UK). Mean comparisons were performed by a least significant difference (LSD) multiple comparison test at *α* <0.05. Volatile data were analyzed via an ANOVA *F*-test using the lmerTest package in R statistical software (www.R-project.org/) [21]. Mixed effect linear models were fitted individually for each volatile, with the response variable being the concentration at each treatment level. A fixed effect predictor was included for treatment, together with a random intercept for wine, to account for the repeated measures on each wine. The fitting was performed using the lme4 package in R [22]. Two statistical analyses were employed using XLSTAT (version 2015.4.1, Addinsoft, NY, USA): (i) a chi-square goodness of fit test, to determine statistical significance of the observed distribution of sample preferences vs. random choice; and (ii) a one sample proportion test, to determine statistical significance between the preferred sample, i.e., the alcohol sweetspot, vs. random choice.

## 3. Results and Discussion

### 3.1. Influence of Partial Dealcoholization by Ro-Ep on Wine Composition

Basic compositional parameters, i.e., alcohol, residual sugar, density, glycerol, pH, TA, VA, organic acids and color, for wines A and B (and their corresponding RAWs) are reported in Table 1 Decreases in alcohol content of 1.6 and 1.8% abv were achieved by RO-EP treatment of wines A and B, respectively. Statistically significant increases in residual sugar levels were observed, but these were not considered meaningful given both wines were dry, i.e., contained <1 g/L of sugar. Similarly, slight increases in density and glycerol were not considered to be meaningful. In the case of glycerol, concentration increases might be explained by its molecular weight (180 atomic mass units, amu), being close to the molecular weight cut-off of the membrane (180–220 amu), such that glycerol was retained during dealcoholization and concentrated with ethanol removal [3]. Anecdotal evidence suggests that for commercial scale dealcoholization (i.e., at the 20–30 kL scale employed in the current study), for every 1000 L of ethanol removed, wine volume decreases by 900 L due to the mixing phenomena of ethanol and water. There were no significant changes in pH, TA or VA following dealcoholization, and with the exception of lactic acid, which increased in concentration by 26% due to RO-EP treatment of wine B, there were no significant changes in organic acid levels, in agreement with previous studies that suggest wine is a well-buffered system [23,24].

Wine color intensity increased in RAW A compared to wine A, while L* (lightness) decreased, indicating color became darker, and a* and b* increased, indicating intensification of red and yellow hues. Significant differences in color were not observed between wine B and its corresponding RAW. According to the literature, changes in color (ΔE*ab) ≥3.0 can be readily distinguished [25]. In the current study, ΔE*ab values were 0.7 and 0.2 for wine/RAW A and B respectively, meaning color changes would not be visually perceptible. Nevertheless, color changes could be explained by a combination of increased anthocyanin concentrations and the formation of copigmented/polymeric anthocyanin complexes, as a consequence of wine concentration with ethanol removal. Previous research has shown that 2% ethanol removal from wine by nanofiltration or reverse osmosis resulted in increases in color intensity of 5.5 and 11%, respectively [26].

In terms of aroma and flavor, partial dealcoholization of wine by RO-EP can affect the concentration of volatile compounds in three ways: i) volatiles can be lost during processing as a result of their passage into the waste stream; ii) the concentration of volatiles can increase in the headspace of wine as ethanol removal affects their solubility and volatility (i.e., ethanol acts essentially as a solvent); and iii) changes in ethanol levels may positively or negatively influence the concentration of volatiles that exist in equilibrium with ethanol [3,27,28], e.g., alcohols, acids and esters. In the current study, ethyl and acetate ester concentrations typically decreased with dealcoholization, in some cases by as much as 50–80% (Table 2); albeit 2-phenylethyl acetate levels increased substantially with dealcoholization of wine A. In contrast, 2-phenylethanol concentrations increased slightly, whereas 1-hexanol levels increased following RO-EP treatment of wine A, but decreased in wine B. Whereas RO-EP treatment was only found to have significantly impacted the concentrations of ethyl hexanoate, ethyl 2-methylpropanoate and ethyl 3-methylbutanoate, this likely reflects the limited sample size (i.e., *n* = 2) available for statistical analysis. Nevertheless, where dealcoholization results in sub-threshold concentrations of volatiles (derived from either grapes or fermentation), this may diminish the perception of wine aroma and flavor.

### 3.2. Influence of Presentation Order on the Outcomes of Alcohol Sweetspotting Trials

In sensory analysis, it is usual for samples to be presented in a randomized order, so as to minimize bias that might favor certain outcomes over others [17]. It has been suggested that the methodological approach used by industry during alcohol sweetspotting trials, i.e., the evaluation of samples in ascending or descending order of ethanol concentration, might influence panelists’ decision-making [6]. For example, even where the alcohol content of samples is not disclosed, panelists who are familiar with the evaluation of samples comprising incremental changes in ethanol content may tend to avoid selection of sample extremities, i.e., the ‘end’ samples with the lowest and highest ethanol levels, potentially biasing their alcohol sweetspot determination. A key aim of this study was therefore to evaluate to what extent the way in which samples are presented (i.e., in linear (row) vs. circular and randomized vs. ordered formats) might influence the outcome of alcohol sweetspotting trials. The ease with which individual panelists, as well as the panel as a whole, could reproduce sweetspot determinations (using the four presentation formats) was also evaluated. Samples presented in a linear format were tasted from left to right (being from highest to lowest alcohol content), and back again, because the panel (predominantly comprising winemakers with alcohol sweetspotting experience) indicated they felt this approach facilitated the perception of changes within brackets of samples, particularly for palate attributes such as acidity, hotness and astringency. Samples presented in a circular format were tasted in a clockwise direction (starting with a randomly selected sample), and back again.

**Figure 2:**
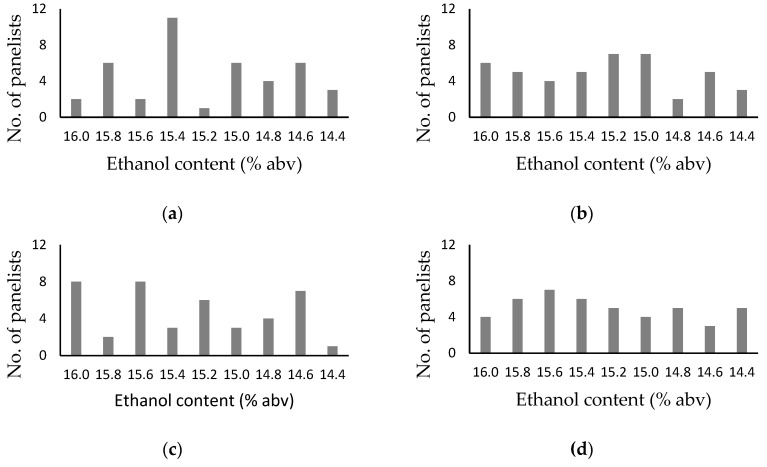
Histograms showing sample preferences from alcohol sweetspotting trials (performed in duplicate, with an expert panel of 14 winemakers) with samples comprising wine A, RAW A and blends thereof, spanning ethanol levels from 14.4 to 16.0% abv, using: (**a**) row, randomized, (**b**) row, ordered, (**c**) circular, randomized and (**d**) circular ordered, presentation formats.

In Trial 1, panelists were permitted to identify one or more samples they considered to exhibit superior overall sensory qualities. The 15.4% blend of wine/RAW A from the row, randomized format was the only apparent sweetspot (Figure 2), with both chi-square goodness of fit and one proportion tests confirming sample preference within this bracket was statistically significant (Table 3). However, this sample was not consistently identified as a sweetspot. Wine A and its 15.6% blend were chosen more frequently than other samples when a circular, randomized format was used for evaluation (Figure 2c), whereas for samples evaluated according to alcohol content (i.e., an ordered format), panelist preferences were distributed across each bracket (i.e., with 4 to 7 preferences given to 7 of 9 samples, Figure 2b,d). Statistical analyses confirmed there were no significant differences amongst these brackets (Table 3). When the outcomes of sweetspotting trials were compared as individual brackets (Figure S1), it can be seen that the 15.4% blend of wine/RAW A was only identified as a sweetspot in one bracket replicate (Figure S1a). Furthermore, when the performance of individual panelists was considered (Table S2), only 16 of the 56 trial 1 sweetspot determinations yielded the same preferences. This suggests panelists found it difficult to identify samples that clearly exhibited superior overall sensory properties, either individually or collectively, and irrespective of presentation format. Disagreement within a panel (i.e., between panelists) might be explained by differences in winemakers’ perceptions of what constitutes ‘superior sensory properties’ (i.e., stylistic preferences), but disagreement in individual panelist’s determinations suggests differences in wine sensory properties as a consequence of compositional changes achieved by partial dealcoholization (Table 1 and Table 2), but ethanol removal in particular, could not be reproducibly discriminated via alcohol sweetspotting.

**Figure 3:**
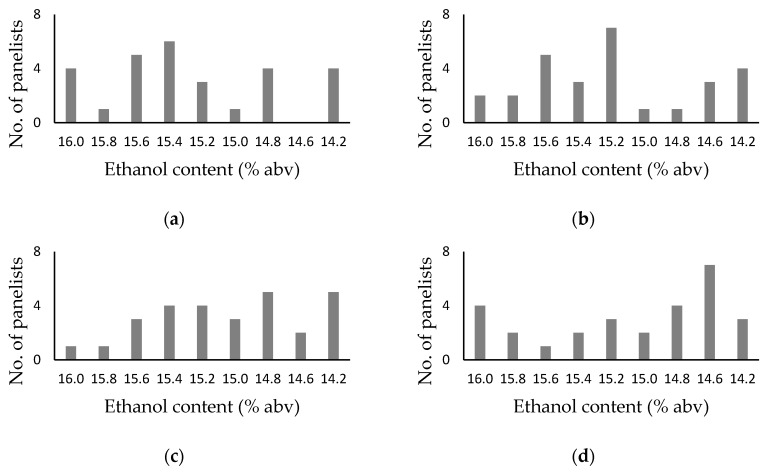
Histograms showing sample preferences from alcohol sweetspotting trials (performed in duplicate, with an expert panel of 14 winemakers) with samples comprising wine B, RAW B and blends thereof, spanning ethanol levels from 14.2 to 16.0% abv, using: (**a**) row, randomized, (**b**) row, ordered, (**c**) circular, randomized and (**d**) circular ordered, presentation formats.

In Trial 2, panelists were only permitted to identify one sample, i.e., the sample considered to exhibit superior overall sensory qualities. Chi-square goodness of fit tests indicated that irrespective of presentation format, the distribution of sample preferences observed for wine B, RAW B, and blends thereof (Figure 3) were not statistically significant (Table 3). However, the 15.2 and 14.6% blends of wine/RAW B from the row, ordered and circular, ordered formats respectively (Figure 3b,d), each received 7/28 preferences, which one proportion tests deemed statistically significant (*p* = 0.030, Table 3). Again, the outcomes of sweetspotting trials were not consistent across individual brackets (Figure S2); only RAW B and the 14.6% blend of wine/RAW B received statistically significant numbers of preference (being 5/14, *p =* 0.014), in one circular, randomized bracket and one circular, ordered bracket, respectively (Figure S2f,h). When the performance of individual panelists was considered (Table S3), only 7 of the 56 trial 2 sweetspot determinations yielded the same preferences; all of which were from ordered presentation formats.

A study investigating the influence of tasting order on the perception of red wines with varying levels of alcohol (from 12 to 16% abv) found alcohol concentration affected wine perception, with panelists who assessed wines in order of decreasing alcohol less able to discern differences amongst wines than panelists who assessed wines according to ascending alcohol levels [30]. The authors suggested sensory differences might be more strongly contrasted between lower and higher alcohol wines (rather than between higher and lower alcohol wines). In the current study, it is unclear if the moderate differences in alcohol concentration (i.e., 0.2% abv) were simply too small to perceptively impact wine sensory properties, or if the order of sample presentation, either randomized, or from highest to lowest alcohol content (and back again), did not sufficiently contrast any sensory differences amongst samples of different ethanol concentrations. Sensory differences may have been more apparent if samples had instead been presented in order of lowest to highest alcohol content and/or if the incremental difference in alcohol content between samples had been larger, albeit this might also have increased the potential for alcohol sweetspots to be missed (i.e., where a sweetspot exists at an alcohol concentration that falls between the incremental change in ethanol content).

### 3.3. Influence of Ethanol Content on the Outcomes of Difference Tests

Triangle tests were performed with wine C and RAW C blends to determine the change in ethanol concentration required to give perceptible differences in wine sensory properties. Although the number of correct responses increased as the difference in alcohol content between wine C and its blends increased (Table 4), the number of correct responses required to establish differences at acceptable levels of confidence (i.e., 10, 12 or 13 for 95, 99 and 99.9% confidence, respectively [17]) were not achieved. Only 5 or 6 correct responses were obtained for brackets comprising RAW C and its blends, suggesting the differences in alcohol content for these samples did not appreciably influence their sensory properties. Informal feedback from panelists indicated these samples were quite astringent, which may have confounded their detection of sensory differences attributable to variation in ethanol content. The panel’s inability to discriminate samples which differed in ethanol concentration by as much as 1.0% was consistent with a previous study that reported ethanol difference thresholds for red wine of 1.08 to 1.32% abv [12]. Sensitivity to ethanol differences have also been shown to depend on wine style, evaluation mode (i.e., orthonasal vs. retronasal) and the assessor (i.e., wine experts vs. consumers) [12,15]. This may further explain the difficulty in ascertaining clear alcohol sweetspots from the sweetspotting trials described above, despite the use of an expert panel, as well as the lack of scientific evidence in support of the alcohol sweetspot phenomenon, more broadly.

## 4. Conclusions

Partial dealcoholization of wines A and B decreased alcohol levels by 1.6 and 1.8%, respectively, with no significant changes to key compositional parameters such as pH, TA, VA or color, but some loss of ethyl esters which could affect wine aroma. Chi-square goodness of fit and one proportion tests indicated preference data were statistically significant for some sweetspot determinations, with the one proportion test discriminating preferences for individual samples relative to random choice, thereby improving the likelihood of statistically significant preferences being identified. However, the outcomes of sweetspotting trials could not be replicated (either by panel or by individual panelists), suggesting identification of samples comprising superior sensory properties was challenging for this set of samples, irrespective of presentation order. It is unclear to what extent this can be attributed to the absence of perceptible differences in sensory properties due to moderate differences in (incremental) ethanol concentrations and/or the order of sample presentation, vs. the non-existence of alcohol sweetspot phenomena. Regardless, the global wine industry employs various strategies to adjust the alcohol content of wine in response to environmental, financial and market challenges, and it is likely that winemakers will continue to use alcohol sweetspotting practices to inform decisions regarding dealcoholization. The use of statistical analyses such as one proportion tests are therefore recommended, to validate any significance of outcomes from alcohol sweetspotting. Future research involving paired comparisons of samples could also be undertaken as an alternate approach to discrimination of samples that exhibit optimal overall sensory properties (i.e., to facilitate identification of alcohol sweetspots, if the alcohol sweetspot phenomena does exist).

## Figures and Tables

**Table 1:** Composition of wines A and B before and after partial dealcoholization.

Parameter	Wine A	RAW A	*p*	Wine B	RAW B	*p*
alcohol (% abv)	16.0 a	14.4 b	<0.01	16.0 a	14.2 b	<0.01
residual sugar (g/L)	0.56 a	0.68 b	<0.01	0.5 a	1.1 b	<0.01
density (g/mL)	0.993	0.995	ns	0.993 a	0.996 a	<0.01
glycerol (g/L)	10.7	11.1	ns	10.9 b	11.6 a	0.01
pH	3.7	3.7	–	3.6	3.6	–
TA (g/L)	6.8	6.8	–	6.8	6.9	–
VA (g/L)	0.7	0.8	–	0.7	0.7	–
succinic acid (g/L)	1.4	1.5	ns	1.5	1.5	ns
lactic acid (g/L)	1.9	2.0	ns	1.9 b	2.4 a	0.01
malic acid (g/L)	0.20	0.23	ns	0.15	0.10	ns
tartaric acid (g/L)	2.6	2.8	ns	2.8	2.7	ns
wine color density (au)	12.4 a	12.6 b	<0.01	15.4	15.4	ns
wine hue	0.7	0.7	–	0.7	0.7	–
L*	62.3 a	61.8 b	<0.01	55.7	55.7	ns
a*	35.5 a	35.9 b	<0.01	41.4	41.4	ns
b*	2.77 a	2.89 b	0.05	4.6	4.5	ns

Values are means of duplicate measurements (*n* = 2). Standard errors were ≤10%. Values followed by different letters within rows (for each wine) are statistically significant (*p* = 0.05, one-way ANOVA); ns = not significant. RAW = reduced alcohol wine; au = absorbance units.

**Table 3:** *p* values calculated for chi-square goodness of fit and one proportion tests of preference data from alcohol sweetspotting trials; the proportion of preferred samples are shown in brackets.

Presentation Order	Goodness of Fit Test		One Proportion Test	
	Trial 1	Trial 2	Trial 1	Trial 2
row, randomized	0.033	0.227	0.004 (11/41)	0.083 (6/28)
row, ordered	0.791	0.270	0.212 (7/44)	0.030 (7/28)
circular, randomized	0.966	0.434	0.089 (8/42)	0.194 (5/28)
circular, ordered	0.151	0.639	0.229 (7/45)	0.030 (7/28)

**Table 4:** Results from difference tests for blends of wine C and RAW C.

	Ethanol Concentration (% Abv) of Samples Evaluated in Difference Tests					
	16.3 v 16.1 (0.2%)	16.3 v 15.5 (0.5%)	16.3 v 15.3 (1.0%)	15.0 v 14.0 (1.0%)	14.5 v 14.0 (0.5%)	14.2 v 14.0 (0.2%)
correct responses	5/18	7/18	9/18	5/18	6/18	5/18

**Table 2:** Volatile composition of wines A and B before and after partial dealcoholization.

Volatile Compound	Descriptors	Threshold	Wine A	RAW A	Wine B	RAW B	*p*	SD
ethyl propanoate	fruity	1840	170	75	251	88	ns	3
ethyl butanoate	acid fruit	20	94	21	136	32	ns	1
ethyl hexanoate	green apple	5	11	3	13	5	<0.01	6
ethyl octanoate	sweet, soap	2	7	3	8	6	ns	3
ethyl decanoate	soap	200	2	1	2	2	ns	5
1-hexanol	green, grass	4000	1950	2265	2132	2096	ns	10
2-phenylethanol	roses	10	381	399	376	399	ns	0.7
ethyl 2-methylpropanoate	fruity	15	115	14	95	40	<0.05	4
ethyl 2-methylbutanoate	sweet fruit	1	13	2	14	5	ns	1
ethyl 3-methylbutanoate	berry	3	20	4	22	8	<0.05	0.3
2-methylpropyl acetate	banana, fruity	1600	27	5	38	11	ns	2
3-methylbutyl acetate	banana	30	255	110	723	136	ns	20
2-methylbutyl acetate	banana, fruity	1600	93	35	231	46	ns	5
2-phenylethyl acetate	floral	250	247	739	698	439	ns	1
hexyl acetate	sweet, perfume	670	1	1	7	1	ns	0.1

Concentrations are µg/L, except for 2-phenylethanol which was mg/L. Aroma descriptors and thresholds obtained from the literature [29]. Standard deviation (SD) based on calibration as analyses were not replicated (i.e., *n* = 1). *p* values were calculated from an ANOVA of the likelihood ratio test of a treatment effect (*p* = 0.05).

## Supplementary Materials

The following are available online at https://www.mdpi.com/2304-8158/8/10/491/s1, Figure S1: Histograms comparing sample preferences from replicated of alcohol sweetspotting trials comprising wine A, RAW A and blends thereof, spanning alcohol levels from 14.4 to 16.0% abv, using: (a,b) row, randomized, (c,d) row, ordered, (e,f) circular, randomized, and (g,h) circular, ordered presentation formats; Figure S2: Histograms comparing sample preferences from replicated of alcohol sweetspotting trials comprising wine B, RAW B and blends thereof, spanning alcohol levels from 14.2 to 16.0% abv, using: (a,b) row, randomized, (c,d) row, ordered, (e,f) circular, randomized, and (g,h) circular, ordered presentation formats; Table S1: Demographics and experience of expert panelists; Table S2: Panelist sample preferences from alcohol sweetspotting trials comprising wine A, RAW A and blends thereof, spanning alcohol levels from 14.4 to 16.0% abv, for each presentation format; Table S3: Panelist sample preferences from alcohol sweetspotting trials comprising wine B, RAW B and blends thereof, spanning alcohol levels from 14.2 to 16.0% abv, for each presentation format.
