# Supplementary material for: Investigating Alcohol Sweetspot Phenomena in Reduced Alcohol Red Wines
Source: Foods. 2019 Oct 14;8(10):491. doi: 10.3390/foods8100491 (PMC6835537; doi:10.3390/foods8100491)
Supplement: Supplementary file 1 [file foods-08-00491-s001.pdf]

Supplementary Materials

Investigating Alcohol Sweetspot Phenomena in Reduced Alcohol Red Wines

Duc-Truc Pham [1,2], Vanessa J. Stockdale [2,3], David W. Jeffery [1,2], Jonathan Tuke [4] and Kerry L. Wilkinson [1,2,*]

[1] School of Agriculture, Food and Wine, The University of Adelaide, Waite Campus, PMB 1, 5064 Glen Osmond, Australia; duc-truc.pham@adelaide.edu.au (D.-T.P.); david.jeffery@adelaide.edu.au (D.W.J.)
[2] The Australian Research Council Training Centre for Innovative Wine Production, PMB 1, 5064 Glen Osmond, Australia; Vanessa.Stockdale@tweglobal.com
[3] Treasury Wine Estates, 97 Sturt Highway, 5352 Nuriootpa Australia
[4] School of Mathematical Sciences, The University of Adelaide, 5000 Adelaide, Australia; simon.tuke@adelaide.edu.au
* Correspondence: kerry.wilkinson@adelaide.edu.au; Tel.: +61-8-8313-7360

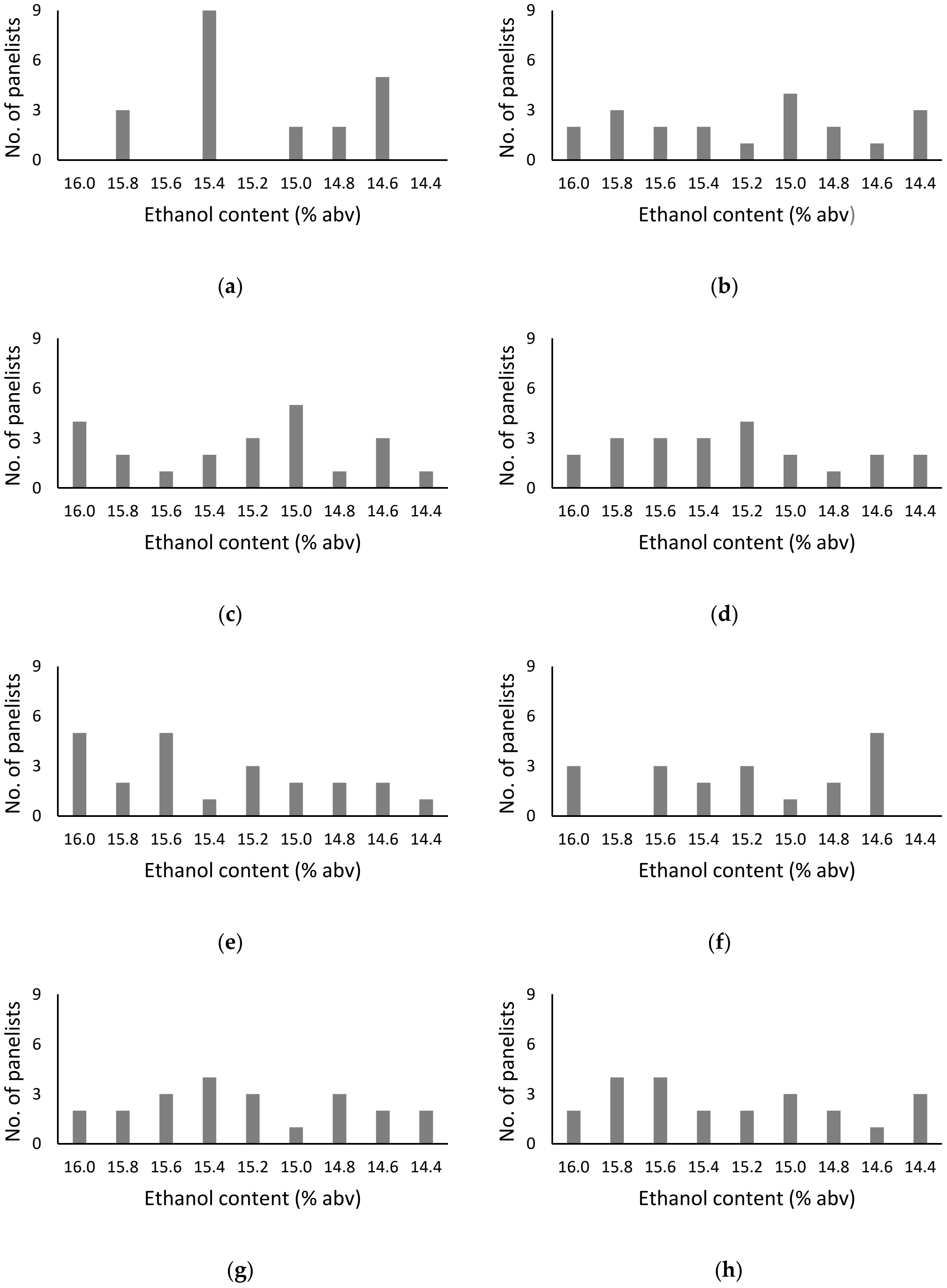

Figure S1. Histograms comparing sample preferences from individual replicates of alcohol sweetspotting trials comprising wine A, RAW A and blends thereof, spanning alcohol levels from 14.4 to 16.0% abv, using: (**a**,**b**) row, randomized, (**c**,**d**) row, ordered, (**e**,**f**) circular, randomized, and (**g**,**h**) circular, ordered presentation formats.

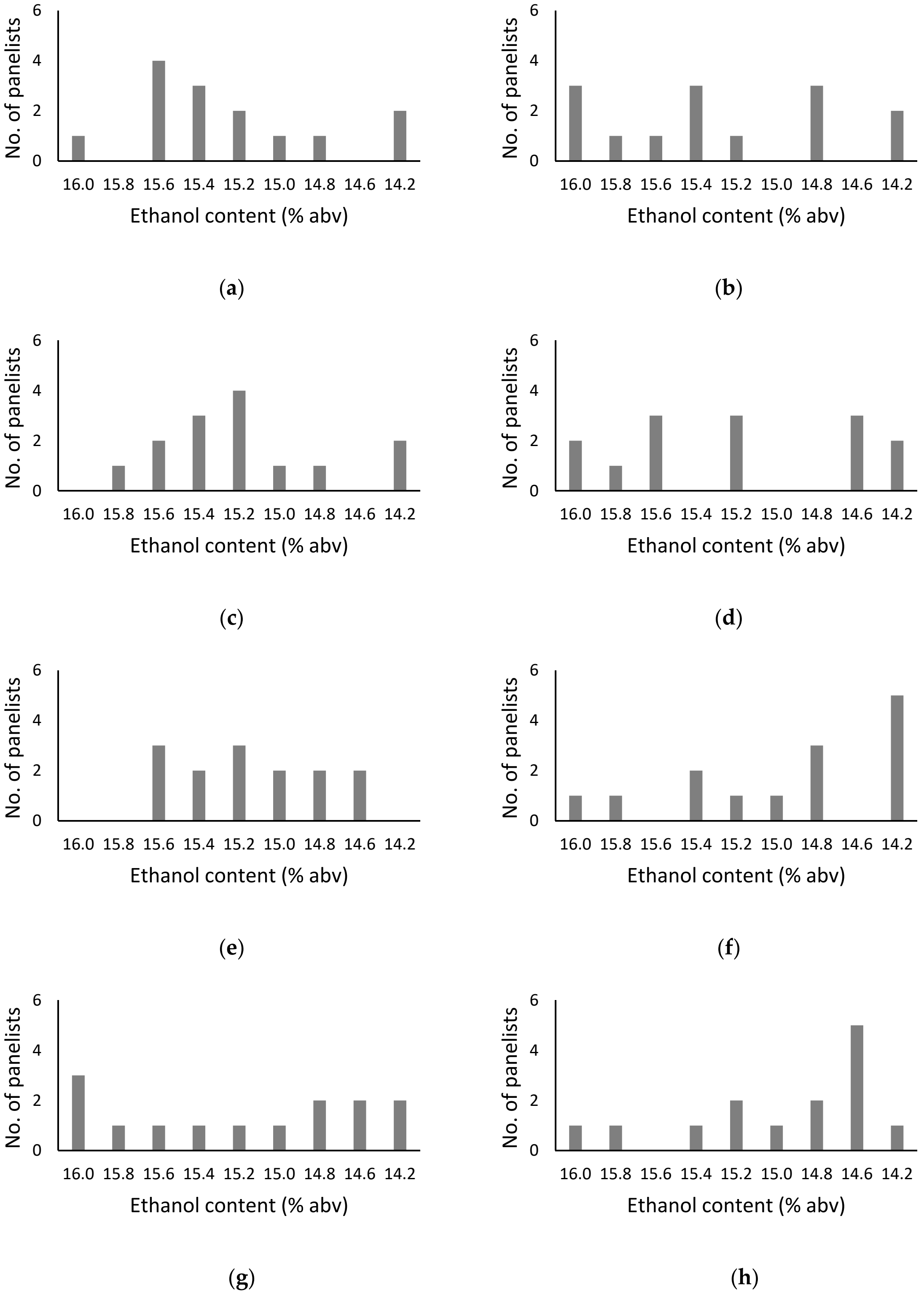

Figure S2. Histograms comparing sample preferences from individual replicates of alcohol sweetspotting trials comprising wine B, RAW B and blends thereof, spanning alcohol levels from 14.2 to 16.0% abv, using: (**a**,**b**) row, randomized, (**c**,**d**) row, ordered, (**e**,**f**) circular, randomized, and (**g**,**h**) circular, ordered presentation formats.

Table S1. Demographics and experience of expert panelists.

	**Trial 1 (n=14)**	**Trial 2 (n=14)**
Sex		
female	21	36
male	79	64
Age (years)		
≤ 30	7	7
31–40	43	64
41–50	29	21
≥ 51	21	7
Wine industry experience (years)		
≤ 10	7	28
11–20	58	58
≥ 21	35	14
Wine judging experience?		
Yes	100	100
No	0	0
Alcohol sweetspot experience?		
Yes	79	79
No	21	21

Data are presented as percentages.

Table S2. Panelist sample preferences from alcohol sweetspotting trials comprising wine A, RAW A and blends thereof, spanning alcohol levels from 16.0 to 14.4% abv, for each presentation format.

Presentation format	Panelist													
	1	2	3	4	5	6	7	8	9	10	11	12	13	14
row, randomized 1	8	8,2	2,4	4, 7	4,2	4	4	4	8,4	6	4,8	8,6	4	7
row, randomized 2	9	1,3	1,9	7	2	4	2	6	4,8	2	6	9,6	7,3,5	6
row, ordered 1	5	3,6	2,8	4	1,6	1,5	1	5,6	8,7	6	4	6,8	1,9	2
row, ordered 2	3	5,4,6	2,8	5	2	3,9	9	7	1,5	4	5,8	6,3	1,2	4
circular, randomized 1	1	7,3	7	2,6,4	5,1	1,9	2	5	1,3	3	1,3	6,8,5	8	3
circular, randomized 2	5	3	1,5	4,8	3	8	8	4	7,8	1	7	6,3	1,8	5
circular, ordered 1	7	7	3,9	1,6	2,4	9	4	4,5	1,4	8	8,7	3,5	3,2	5
circular, ordered 2	6	4	6,9	7,2,5	3	2	2	7	9,5	1	2,3	1,3,8	4,9,3	6

Sample preference numbers (i.e., 1 to 9) correspond to decreasing ethanol concentrations (i.e. 16.0 to 14.4%).

Table S3. Panelist sample preferences from alcohol sweetspotting trials comprising wine B, RAW B and blends thereof, spanning alcohol levels from 16.0 to 14.2% abv, for each presentation format.

**Presentation format**	**Panelist**													
	**1**	**2**	**3**	**4**	**5**	**6**	**7**	**8**	**9**	**10**	**11**	**12**	**13**	**14**
row, randomized 1	3	5	3	1	4	5	4	3	6	9	3	4	9	7
row, randomized 2	4	3	1	4	9	7	9	1	7	7	5	1	2	4
row, ordered 1	4	7	3	3	5	5	9	4	5	4	6	2	5	9
row, ordered 2	3	9	5	2	3	8	8	3	5	8	9	1	5	1
circular, randomized 1	7	6	4	8	3	4	3	5	5	3	8	5	7	6
circular, randomized 2	9	4	1	9	9	9	9	2	7	5	7	7	6	4
circular, ordered 1	6	9	7	7	8	9	4	3	5	2	8	1	1	1
circular, ordered 2	8	8	7	6	2	9	4	8	5	7	5	8	8	1

Sample preference numbers (i.e., 1 to 9) correspond to decreasing ethanol concentrations (i.e. 16.0 to 14.2%).
